# Dactylitis Syphilitica

**Published:** 1890-02

**Authors:** J. C. Olmsted

**Affiliations:** Atlanta, Ga.


### DACTYLITIS SYPHILITICA.

BY J. C. OLMSTED, M. D., ATLANTA, GA..

With remarks on the Nature of the Disease and Report of a Case.

I have thought that the report of a case of this rather rare disease, might be of interest, and not without practical importance, as the necessity of its *recognition*, and *proper treatment*, was strongly illustrated by this case. As shown in this history, the *rarity* of the affection is no security that *any* of us may not be confronted with a case of it; and mistakes and consequent mortification may be avoided by a knowledge of its nature and familiarity with its diagnostic signs.

H. F., male, white infant, aged twenty-two months, was brought to me by Dr. F., in October, 1888, for an opinion as to the nature of an affection of his finger. The history given by the mother was that she had "noticed for some time" a marked and peculiar enlargement of the ring finger of the right-hand, which enlargement had developed slowly, was *unaccompanied* by any *discoloration* of the *skin*, signs of *pain* on pressure, or otherwise, or indeed any evidences of *inflammation*. It attracted attention only by its extraordinary size and consequent deformity, which led the parents to finally seek medical advice. Three or four physicians were consulted, two of them being quite distinguished practitioners. All of them admitted that it was "a peculiar case," and one of the latter stating that he had "never seen anything like it in his life," advised her to seek the services of a distinguished surgeon, of this city, which she immediately did. This gentleman, after a rather cursory examination of *the finger*, promptly told her that the finger "would have to *come off*." When asked if *medicine* would not "cure it" he replied that "a *barrel* of medicine would do no good," and that "*amputation* is the only remedy." The mother left with her child, and in great mental distress at this verdict; meeting Dr. F., casually, she stated the case, and he expressing dissent, advised her to bring the child to me; coming with her himself, he introduced the little patient, *expressing no opinion*, but desired me to give mine' 'perfectly unbiased." Examination of the finger showed a peculiar enlargement of the first phalanx of the ring finger of the right-hand. This was most peculiar, giving the entire finger the shape somewhat of a "nine-pin." The skin was perfectly natural in color, there being no redness, or discoloration of any kind. Free manipulation of the finger failed to elicit any symptoms of pain. The joint was not involved, although embarrassed in its free movements by the ridge formed by the bulging border of the swelling, which ended abruptly at the joint. The swelling itself was firm and resistant, involving the entire circumference of the phalanx, being most prominent on its dorsal surface, no pitting upon pressure was developed. In the largest part of the swelling, the circumferential measurement was four and a quarter inches The other fingers of this hand were natural. The other or left hand showed enlargement of the first and second phalanges of the ring finger, with a small ulcer on the palmar surface. The enlargement of the bones was limited, and the finger seemed to be undergoing contraction. The mother informed me that this finger had been more swollen, than at this time, but nothing in comparison with the finger of the other hand. The small ulcerated surface mentioned, was over the joint, on the palmar aspect, and in the line of the flexor tendon; it was quite superficial and sluggish in appearance, being free from any appearance of active inflammation. I next directed my attention to the child's teeth, and found the notched central incisors so much esteemed by Mr. Jonathan Hutchison, as diagnostic of hereditary syphilis. I also found induration of the cervical, post cervical, and occipital glands.

The cervical glands were so much enlarged as to remind me of strumous lymphadenitis. The remarkable size of the child's head impressed me. The shape was of the hydrencephaloid type. I found the circumferential measurement to be twenty-two inches. Large, lustrous, dark eyes with long, curling lashes, a thin skin with delicate coloring, combined with a remarkably precocious and interesting manner, to suggest again the strumous type of constitution. The child appeared fairly well nourished, and, although I examined his whole body, (stripping him for that purpose), there was nothing of further interest observed, saving that one or two *toes*, on each foot, showed a slight but distinct tendency to the same kind of enlargement which existed on the hands. Cautious questioning of the mother, who was a refined lady, elicited the fact that the baby "had had some spots, or sores, on his body" a few days after he was born. She could not say whether there were any upon him *when* he was *born* or not; she noticed them a day or two after his birth. Also, he had "little crops of sores, come and go," at intervals since. Questioned as to her own health prior to the birth of the child, she said that she had been "in poor health," but nothing definite, or even significant of specific disease, was elicited by these questions. She was a delicate, pale, spare-looking woman, the mother of four children. She was allowed to withdraw, comforted by the assurance that her baby's finger would *not* have to be *amputated*; and was also told that I would speak to her *husband* fully in regard to the case. To Dr. F. I gave my diagnosis as being "*Dactylitis Syphilitica*," of hereditary form; in which he fully concurred. By his advice the patient was turned over to me for treatment, and the father of the child was frankly informed by me of my views concerning the cause of the disease. With professed frankness he denied all contagion through himself; "never had been afflicted with any disease of that sort," etc. His wife's character I *knew* to be beyond suspicion. I held to my opinion, however, and told him I should *treat* the *child* for *this disease*. He readily assented, and seemed much gratified at my positive views as to what was required. The child was placed upon the treatment for tertiary syphilis—mercury and iodide of potassium being the remedies employed. The biniodide of mercury was the form in which the mercury was administered. This was given in small doses, gradually increased. Potassium iodide was given in combination. Mercurial ointment was applied to the enlarged finger, and kept in contact with it under the pressure of plaster, with which the finger was strapped. This treatment was kept up for months, being suspended at intervals (as during the past summer) when the child's bowels were irritated. Cod liver oil and the hypophosphites (Churchill's formula) have also been used with advantage in this case, especially at intervals when the specific medication was suspended. During these intervals of suspension, I may remark that from time to time crops of late eruptions of syphilis occurred, chiefly of the furuncular and pustular varieties—papular syphilis of the flat variety, and scattered sparsely over the extremities, were also observed. I am also satisfied that the child was of strumous diathesis, in addition to being infected with syphilis. The marked tendency to affections of the lymphatic ganglia, which was from time to time shown by marked enlargement of those glands, as well as the general physiognomy of the child, pointed strongly towards this diathesis.

At the present writing the child is in very good health. His greatly enlarged finger, while not entirely natural in size, is nearly so. The gummous material which caused it, having undergone a gradual absorption, without there being at any time ulceration, or other open lesion. The ulcerated surface of the other finger healed quite promptly, a small indentation on the palmar surface, showing where some loss of tissue had occurred. The child is of a very nervous temperament, and very restless, even while sleeping. I expect trouble from tha brain of his yet; he is remarkably precocious and intelligent for his age.

As regards this disease "Dactylitis Syphilitica" for which he was originally brought to me, the compass of this article will forbid my fully discussing the subject. I will briefly state that this disease is *one* of the many manifestations of Tertiary Syphilis. The term "Dactylitis" is from a Greek word, signifying "digit," and is applied in this affection to both fingers and *toes*, according as either are affected. This disease is caused both by acquired and by hereditary syphilis. It is in its nature a gummous formation. There are two varieties. In the first or superficial form, the gummous deposit involves the subcutaneous connective tissue, the fibrous tissues, and soft parts generally. In the second form the bone is attacked, and the condition is that of either specific periostitis, or osteomyelitis. The *proximal* phalanges are most frequently attacked; the distal less often: all three phalanges *may be* involved at the same time. One or more bones of one, or of each hand may be involved. The swelling may progress slowly, or with great rapidity. Frequently the affection runs a slow and very chronic course. The integument often remains unchanged, even where the swelling is very great, (as in my case) or it may become reddened, or violaceous in hue; may inflame actively and ulcerate, discharging its contents, as gummata do elsewhere. Under specific treatment the gummous material will undergo absorption, and the parts may return to their normal condition, with no lesion whatever having occurred. At times joints become involved, the cartilages eroded, false joints, contractions, and other deformities may remain, occasionally requiring surgical interference. This, however, is seldom needed, as it is often surprising how well the parts accommodate themselves where serious deformity was apprehended. Diagnosis is most important, early recognition of the disease preventing destruction of tissue and deformity. The subcutaneous variety may be mistaken for paronychia, but the absence of acute inflammatory symptoms, especially pain, establishes the diagnosis. The absence of pain is quite marked in this affection. When present it is unusually slight, and enables us to exclude most inflammatory disturbances, and gout. Rheumatoid arthritis is essentially a joint affection and is quite painful. It does not usually attack the phalanges, as does Dactylitis. Dactylitis may be confounded with Enchondroma, but this is a hard, well-defined tumor, preferring the palmar to the dorsal surface, (which latter dactylitis very prominently affects.)

In the diagnosis no one sign should, of course, be depended upon. A careful examination of the patient as a whole should be made, and all of the surrounding circumstances be considered. Information from the parents may give some clue at times, (though not in my case particularly.) I think that careful attention to the physical characteristics of this affection, and the history of the case, should save error. The prognosis where early treatment is instituted is good, but the improvement is slow, and occasional relapses take place.

The treatment is, of course, the constitutional treatment of tertiary syphilis, and I know of no better remedies than Iodide of potassium, and the preparations of mercury; locally mercurial ointment and graduated pressure, (especially the latter,) are very useful. Ulcerated surfaces require various stimulating and appropriate applications on general principles.

